# Course of the first month of the COVID 19 outbreak in the New York State counties

**DOI:** 10.1371/journal.pone.0238560

**Published:** 2020-09-02

**Authors:** Anca Rǎdulescu

**Affiliations:** Mathematics, SUNY New Paltz, New Paltz, NY, United States of America; Columbia University, UNITED STATES

## Abstract

We illustrate and study the evolution of reported infections over the month of March in New York State as a whole, as well as in each individual county in the state. We identify piecewise exponential trends, and search for correlations between the timing and dynamics of these trends and statewide mandated measures on testing and social distancing. We conclude that the reports on April 1 may be dramatically under-representing the actual number of statewide infections, an idea which is supported by more recent retroactive estimates based on serological studies. A follow-up study is underway, reassessing data until June 1, using additional measures for validation and monitoring for effects of the PAUSE directive, and of the reopening timeline.

## 1 Introduction

Since its first confirmed infections in the US, it has become clear that the COVID-19 outbreak was going to affect all US states and territories. However, the reported rates of infection, hospitalization and death have been from the start subject to wide controversy that sprouted from the known limitations in testing abilities, potentially resulting in dramatic under-reporting. Failure to accurately report infection rates can crucially alter our assessment of the size and dynamics of the pandemic, and our predictions of its future evolution. It also affects administrative decisions on the type and timeline of necessary social distancing measures. In the absence of precise measurements of infection rates, it is therefore very important, when interpreting the available data on confirmed infections, to differentiate between trends that are specific to the epidemic dynamics (and the effects of social distancing specifications), and artifacts introduced by limitations on testing availability or by reaching medical care capacity. The former are governed by the virus’ clinical characteristics and the interactions in the social network where the virus propagates (in the presence or absence of additional social regulations); the latter are only a reflection of assessment limitations. In this study, we aim to tease apart such patterns.

Overall, there have been notable between-state differences in the timeline and magnitude of the epidemic. It is likely that these differences arise from a combination of inherent factors, from timing of the first infection (earlier states were caught unprepared), to population density and intrinsic social dynamics. Differences may also arise from variations in timing and efficiency of statewide mandated directives (travel bans, social distancing, timing and availability of testing). We work from the premise that patterns resulting from statewide control (1) lead to accentuating between-states differences, and (2) can explain unifying trends in the data from same-state counties, transcending the between-county variability based on inherent factors. Our study investigates this premise based on the first month of data on the epidemic development in the US, focused specifically on its evolution in the State of New York, which, to date, had the highest and fastest climbing infection counts. After a brief comparison of the statewide evolution of confirmed infections with that in other early states, we focus on analyzing patterns unifying New York counties beyond intrinsic variables such as time of first exposure, or population density.

In terms of policy, New York State’s response to the pandemic came in a few stages, both in terms of testing and of mandated social measures. Consistent with the national trend, the testing response had trouble initially scaling up in New York State [1], acquired some momentum, but only for a short period of time [2, 3]. Based on the shortage of protective equipment, a Health Department Advisory capped testing again in the New York City on March 20th: “Outpatient testing must not be encouraged, promoted or advertised[…] There is a national shortage of personal protective equipment (PPE),[…] and it is critical that laboratory testing be prioritized for hospitalized patients.” [4–6]. While not government mandated, similar restrictions on testing were simultaneously implemented in other parts of the state as a wide-spread approach, by both health care providers [7, 8] and mobile testing sites [9, 10].

In terms of social dynamics restrictions, among the first to close in the New York State were university campuses and schools [11, 12], followed by restaurants, gyms and other entertainment venues [13, 14]. During the month of March, gatherings of gradually smaller sizes were banned, all eventually leading to the PAUSE directive that took effect on March 22 and shut down statewide all non-essential activity [15, 16]. When investigating the potential effect of social dynamics on the epidemic spread, it is important to remember that, while changes in testing flow may instantaneously reflect in the number of reported infections, effects of social distancing have a significant lag, due to the incubation period of the virus, and the corresponding time delay between exposure and symptoms.

## 2 Modeling methods

The study of early epidemic growth has historically revealed different patterns, depending on the particular pathogen, and even on the particular outbreak. While other growth patterns have also been found (e.g., polynomial, subexponential), exponential growth has been a seemingly ubiquitous trend detected in data of early outbreaks of influenza, Ebola, foot-and-mouth disease, plague, measles and smallpox [17]. This behavior appears to be driven by the free spread of the pathogen in the first stages of the epidemic [18]. Exponential growth patterns appear to also be representative of the current COVID-19 pandemic, during its early development in the US overall. The first aim of our study is to verify whether this was the case for the total number of confirmed infections in New York, as reported daily for the duration of the first month of the outbreak (March 2020). The second aim is to illustrate that, prior to April 1, the outbreak size and timeline varied significantly between different states. To do this, we chose to briefly compare three of the states with the earliest reported infections of COVID-19, and with the highest infection counts to date: California (9,816 infections by April 1), Washington State (5,588 infections) and New York State (83,948 infections). Thirdly, we aim to focus more specifically on the outbreak dynamics within New York State, to understand both the specific and the unifying trends in the data from different counties, and to correlate these trends with state-mandated measures on testing and social distancing.

### Data sources

The epidemic data for this study was accessed on April 1 from two sources in the public domain: the data-set provided by the Johns Hopkins Center for Systems Science and Engineering [19] and the one maintained by the COVID Tracking Project [20]. The COVID Tracking Project public repository (covering the period March 4 to April 1) was used for the statewide reports of confirmed infections and number of tests, as well as of COVID-19 associated hospitalizations and deaths. The reports provided through the Johns Hopkins Center for Systems Science and Engineering (covering the period March 1 to April 1) were separated by county, and were used for the county-wise analyses and comparisons. We performed a cross-validation of the two data sources on the number of statewide confirmed infections, which was reported by both. On the common portion (March 4 to April 1), the two were identical, except for minor details which are discussed in the Results section. Additional demographic data (census data and population density in each New York county) was obtained from the New York State Department of Health web page [21].

### Data fitting

In order to more easily detect potential exponential growth *N*(*t*) = *N*_0_ *e*^*αt*^ (where *t* is time in days, and *N* is number of infected individuals), we consider the logarithm of the time series ln*N* = ln*N*_0_ + *αt*. This allows to test for linearity and piecewise linearity of the logarithmic time-series by using a simple linear regression algorithm, maximizing the goodness of fit. Piecewise linearity is understood in the context of discrete time series as linearity on each of the *K* consecutive pieces $\left[t_{j_{k}},t_{j_{k+1}}\right]$ that form a partition of the whole time interval $\left[t_{j_{0}},t_{j_{K}}\right]$. For each such piece we calculate the slope *α*; since the pieces vary in length, we compute both the sum of squares and the Pearson *χ*^2^ to illustrate the goodness of fit.

## 3 Results

### 3.1 Exponential growth and between-state comparison

**Fig 1:**
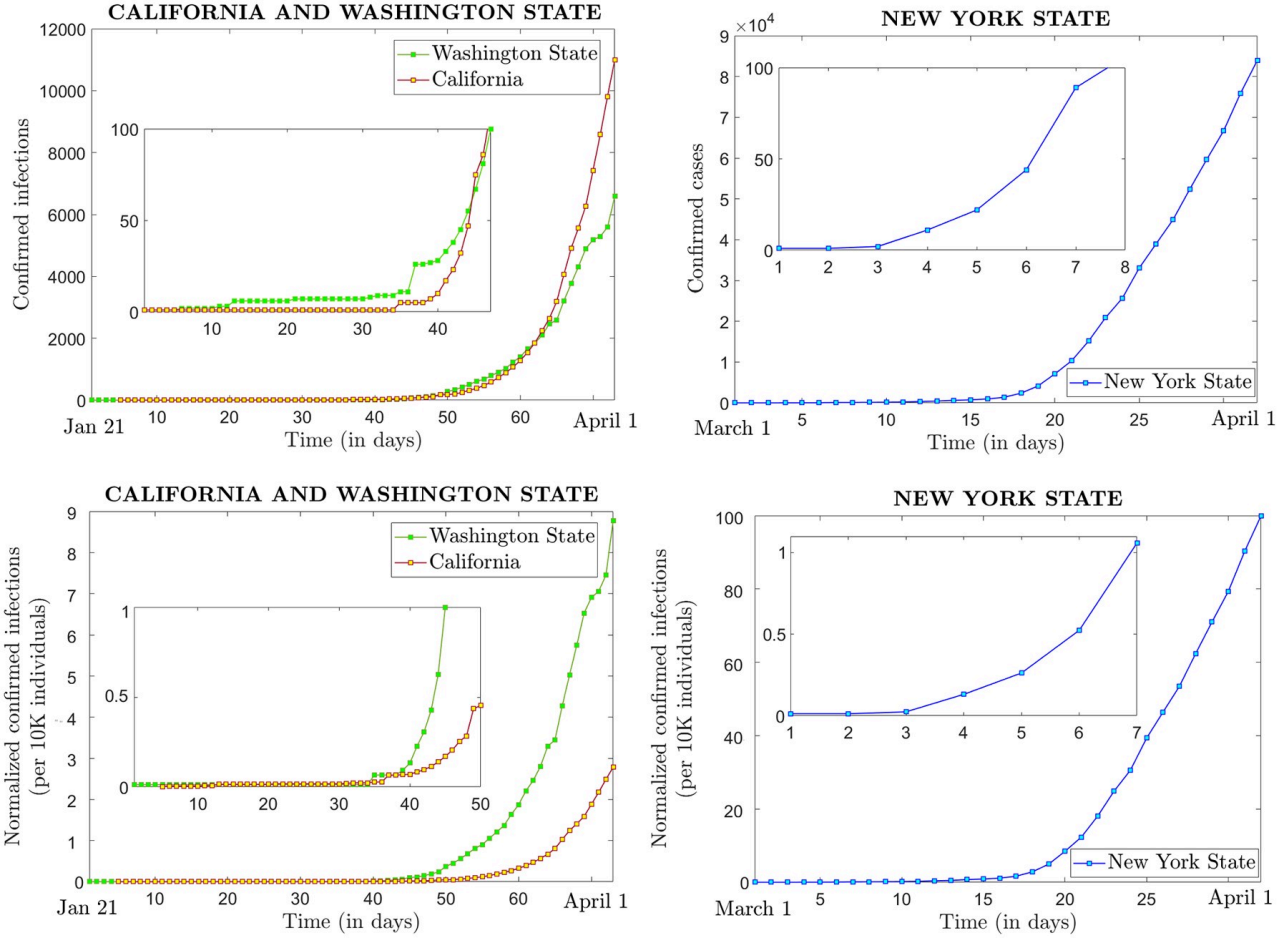
Raw and normalized time series for the states of California, Washington and New York. **Top**. Raw time series for confirmed infections for California and Washington State (left) and New York State (right). On the left, the two time series are illustrated along the same time axis starting on January 21; on the right, the time axis starts on March 1, which marks the first New York State infection. The inserts show details in the early stages of the same time series, up to the point where confirmed infections increased past 100. **Bottom**. Normalized time series for confirmed infections reported per 10,000 individuals, for California and Washington State (left) and New York State (right). The time axes are labeled the same way as in the top panels. The inserts show details in the time series, up to the point where confirmed infections increased past 10 infections/10,000 individuals.

**Fig 2:**
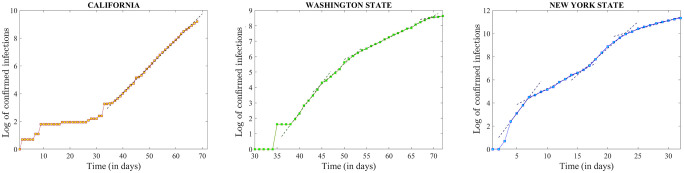
Logarithmic representation of the time series for the states of California (left), Washington (center) and New York (right), illustrating their approximately piecewise linear behavior. The piecewise linear fit is shown as a dashed curve in each case, and the values of the slope and goodness of fit are shown in Table 1.

What one would generally expect to see in the epidemic development is an initial period with high growth rate (potentially exponential). As the growth rate subsides, the time series should move to a more slowly growing curve, then finally reach a peak, and eventually transition to a decreasing trend (as the epidemic is dying out). Figs 1 and 2 illustrate a comparison between time series extracted from the Johns Hopkins data-base [19] for the states of California, Washington and New York. These are all states with early infection compared to other states (January 21 in Washington, January 25 in California, March 1 in New York).

Fig 1 shows the infection timeline for each of the three states, both as raw time series of confirmed infections (in the top two panels), and as number of confirmed infections normalized by the population size in each state (i.e., reported per 10,000 individuals, in the two bottom panels). For each panel, the inserts magnify detail in the first period of the timeline, where the initial small numbers would otherwise be indistinguishable in comparison with the subsequent climb in the graph. California and Washington State had their first confirmed infections four days apart, and had comparable evolutions in terms of the sheer number of infections (although their increasing patterns appeared different). When normalizing by the state population, the curve corresponding to Washington State was significantly higher than that for California (three times as high at the end of the time interval). We chose to represent the state of New York in a separate panel for clarity, because in both instances (of raw and normalized time series), the values of the New York State curves were one order of magnitude higher than for the other two states, by the end of the time interval. We continued by investigating the more subtle trends in rate that may underlie the noticeable differences in these three evolutions.

Fig 2 shows the logarithm of the raw time series, with the day of progression labeled from the start of the outbreak in the respective state. The unifying pattern in this logarithmic representation between the three states is that all three are approximately piecewise linear (with piecewise slopes *α* and goodness of fit shown in Table 1). It also suggests that all three original time series were still growing exponentially in the time interval preceding April 1. It is clear, however, that there are qualitative differences in the dynamic patterns for each state—reflected in the length, succession and slope of the linear pieces. According to this data, the number of confirmed infections in California can be faithfully described by the same exponential curve with slope *α* ∼ 0.18 since day 36 (February 29), with a slight sub-exponential tendency towards the very end of this interval. Starting with day 38 (February 27) Washington follows a logarithmic pattern of successively moving (approximately every week) to a new linear piece with slightly lower slope, thus flattening out to an exponential rate close to zero by the end of March. In contrast, the logarithmic timeline for New York State shows an alternation in slope, even after the initial transient spike in confirmed infections (March 1-4): the first almost-linear piece is steep (March 4-7), followed by a more relaxed increase over the following 10 days (March 7-16), only to launch into another steeper increase (March 16-23), and then another apparent reduction in rate (captured with significant goodness of linear fit over the 10 day segment prior to April 1).

**Table 1:** Rates of exponential growth in the time series for statewide confirmed infections for the states of Washington, California and New York. These were calculated as piecewise slopes of the logarithmic representations of the time series shown in Fig 2. Both sum of squares and the Pearson goodness of fit statistic are also provided in each case.

County	Interval	Slope *α*	Sum of squares	Pearson *χ*^2^
California	36-68	0.1898	0.1528	0.0231
Washington	38-45	0.3842	0.0181	0.0064
	45-52	0.2609	0.0334	0.0067
	52-69	0.1416	0.0146	0.0020
	69-72	0.0418	<10^−4^	<10^−4^
New York	4-7	0.6965	<10^−4^	<10^−4^
	7-16	0.2739	0.0357	0.0067
	16-23	0.4593	0.0691	0.0079
	23-32	0.1518	0.0347	0.0033

From here, we will pursue two directions with our analysis, aimed at understanding whether transitions from one exponential rate to another in the case of New York State are intrinsic to the epidemic dynamics or are a reporting artifact. To this aim, we first test if these transitions correlate temporally with the timeline of statewide directives on social measures or with the testing/reporting schedule. In Section 3.2, we examine state wide data on confirmed infections, hospitalizations and deaths in conjunction with data on testing, for the period from March 4 to April 1, and look for potential triggering events. We need to recall that, while the effects of changes in testing can be observed immediately, the effects of social distancing measures on decreasing infections involve an observation lag that can be as long as a few weeks.

New York State is relatively large geographically, and presents a wide range of social profiles (urban, suburban, rural). The transitions between rates may be driven by specific social behavior in counties with dense population (which have more statistical power), or may be similar across counties with different social profiles (in which case they are more likely to be triggered by statewide mandated measures). In Section 3.3 we analyze the piecewise linear succession in time series for confirmed infections for all New York counties which had reached over 100 infections by April 1, and we look for unifying trends. This will establish whether the slope alternation pattern which appears to be the signature of the evolution of confirmed infections in New York State is an effect of averaging geographically over many types of different evolutions, or if it emerges from a consistent similar pattern across county borders.

### 3.2 Statewide trends

We illustrated and compared the time series for confirmed infections, for hospitalizations and for COVID-19 related deaths versus the statewide number of individuals tested for COVID-19. Since this data was accessed from the COVID-19 Tracking Project repository [20], a different source from that used in Section 1, we first cross-validated the two sources for the one variable they have in common: number of confirmed infections. Logarithmic time series for confirmed infections are illustrated from both sources in Fig 4 (for the period of March 1 to April 1 in the case of the Johns Hopkins archive, and for March 4 to April 1, which was the time interval available in the case of the COVID-19 Tracking Project archive). The graphs show only small differences with no consequence on the overall trends or on our analysis.

**Fig 3:**
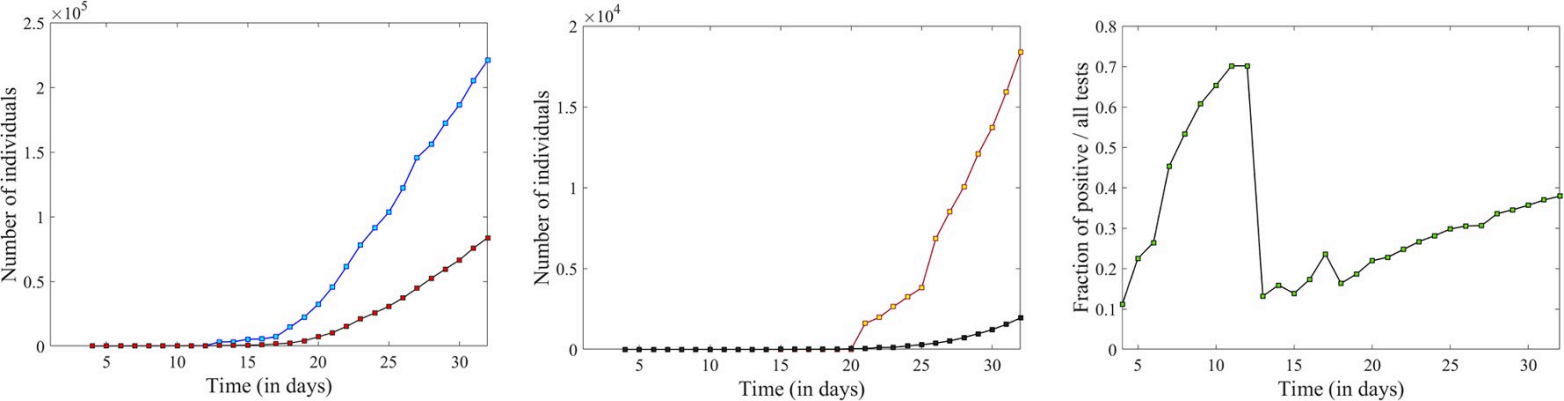
Time series for the total number of administered tests, confirmed infections, hospitalizations and casualties for the New York State, over the period of March 4 to April 1. **A**. Total number of COVID-19 tests administered is shown in blue, and number of positive tests (i.e., number of confirmed infections) is shown in red. **B**. Number of COVID-19 diagnosed hospitalizations in yellow (data missing prior to March 20), and number of COVID-19 associated deaths in black. **C**. Fraction of confirmed positive diagnoses out of the total number of tests performed statewide.

**Fig 4:**
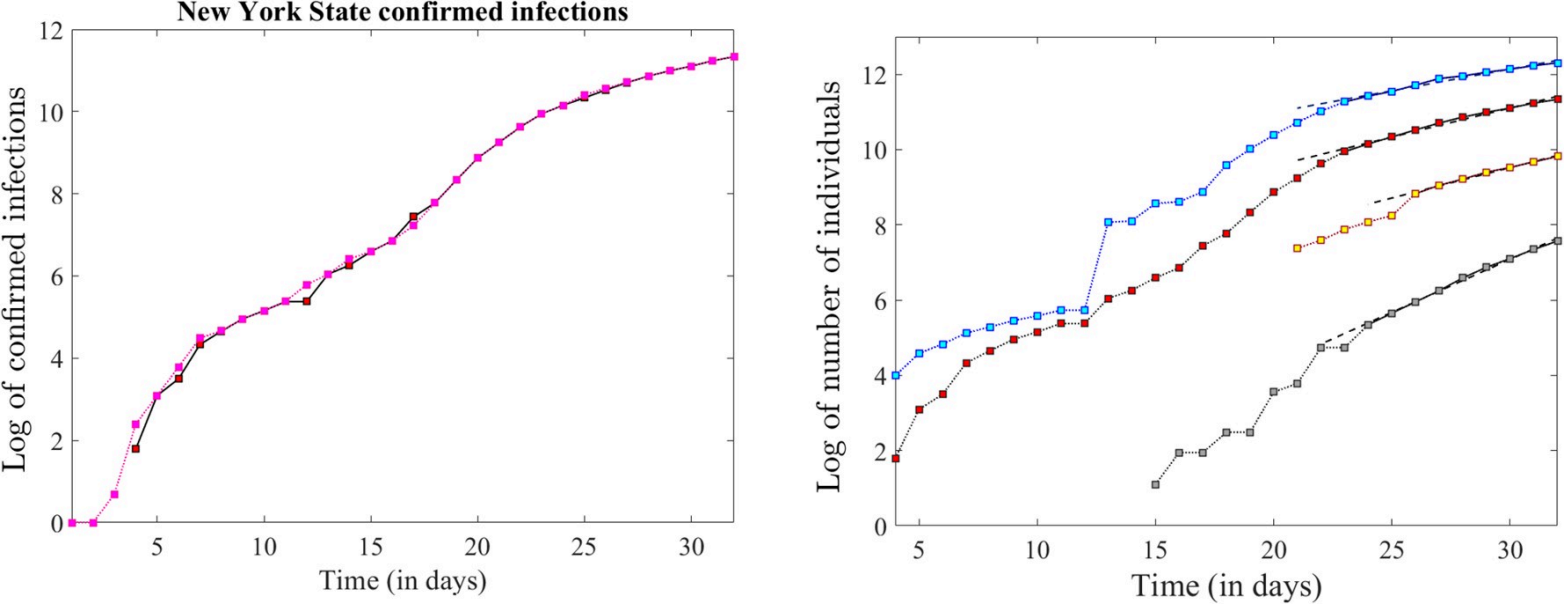
Logarithmic representation of the total number of administered tests, confirmed infections, hospitalizations and casualties. **A**. Comparison of the logarithmic time series of the confirmed infections, as provided by the data source used for this section [20] (in red) and by the date source used in the next section [19] (in pink). **B**. Logarithmic time series corresponding to the raw time series in Fig 3, panels A and B, with the same color coding. The illustration shows all these to be approximately linear over a significant time period before April 1st. The slopes and goodness of linear fit are shown in Table 2.

The numbers of confirmed infections, hospitalizations and deaths, as well as the number of total statewide tests, are shown as raw time series in Fig 3a and 3b, then in logarithmic form in Fig 4b. While the first panel reveals that all variables increased dramatically, and provides a better illustration of their actual size, the latter better conveys the fact that all variables had stabilized along an almost exponential curve of constant rate for over a week prior to April 1 (the exponential fit is quantified in Table 2). The confirmed infections and the death count show other transient rates before this steadier trend. The hospitalizations count is considered more robust epidemiological prevalence measure (since it relies on symptoms, rather than on general testing availability). However, data was only provided in the reference for the last 12 days of March, making it difficult to tell if there were any other consistent trends prior to March 20. In addition, symptomatic COVID-19 infections reportedly exceeded hospital capacities in New York State, associating potential artifacts to this measure as well [22, 23].

**Table 2:** Rate of exponential growth calculated, for each of the variables in Fig 4b, as the slope of the linear fit to the logarithmic time series, on the longest significant interval before April 1. The sum of squares and Pearson goodness of fit statistic are also provided in each case.

Curve significance	Interval	Slope *α*	Sum of squares	Pearson *χ*^2^
Total tests	23-32	0.1155	0.0236	0.0020
Positive tests	23-32	0.1545	0.0257	0.0024
Hospitalizations	26-32	0.1614	0.0048	0.0005
Fatalities	24-32	0.2817	0.0193	0.0029

The time series for total COVID-19 tests performed in New York State shows a notable jump between March 12 and March 13. This spike stands out in the logarithmic form in Fig 4b, and appears even more prominent when representing the fraction of the tests that returned a positive diagnosis (shown in Fig 3c), which plummeted from 70% on March 12 to less than 15% on March 13. This is not surprising, since higher availability of tests can be responsible for a sudden increase in negative results (due to more liberally providing tests to susceptible individuals who turned out to not be infected). The idea will be revisited as a discussion point in Section 4.

### 3.3 County-wise behavior

To study infection dynamics at the county level, we used New York State data from the John Hopkins archive [19], which provided county-wise reports for the period from March 1 (when the first case was reported in the State of New York) to April 1 (the date this study was initiated). County-wise demographic information was obtained from the Department of Health web page [21].

When considering the number of confirmed infections for each county on April 1, one simple observation is that they did not only correlate with the county total population (correlation coefficient *R* = 0.9, significance value *p* < 0.0001), but also with the population density (correlation coefficient *R* = 0.4195, significance value *p* = 0.0007). As a start, this suggests a deeper dependence of infection rates on the type of social dynamics associated to the population density distribution in each county. In our further analysis, we considered only the counties exceeding 100 confirmed infections by April 1 (providing enough data for an adequate assessment).

In the State of New York, the first signs of contagion were detected and recorded in New York City (on March 1), then a few days later in Westchester (March 4), Nassau (March 5), Rockland (March 6), Saratoga (March 7), Suffolk and Ulster Counties (March 8). One month later, on April 1, the leading five counties in infection counts, with over 3,000 infected individuals, were among this list of early foci. New York City, considered as a sum of Bronx, Kings, Queens, New York and Richmond Counties (47,439 infections), Westchester County (10,683 infections), Nassau County (9,554 infections), Suffolk County (7,605 infections), Rockland County (3,321 infections). On the other hand, Saratoga and Ulster Counties reported 122 and 222 infections respectively, by April 1.

We chose to first illustrate and analyze separately these counties with confirmed early infection (before March 10th). These are networks of communities in which the infection had already propagated for a long enough time to provide more substantial data that may allow us to understand the mechanics of this propagation. We will illustrate separately the five counties which had over 3,000 confirmed infections by April 1, and the two counties which, despite an early start, were an order of magnitude lower in the number of confirmed infections by the same date.

**Fig 5:**
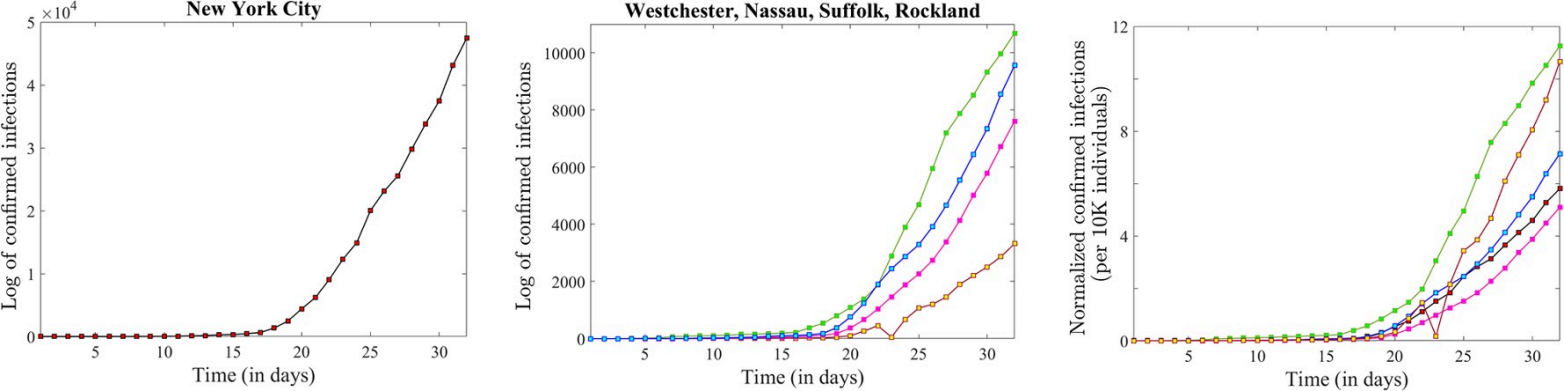
Time series showing the progression in the county-wise number of infected individuals up to April 1, for the counties with early confirmed infections (before March 10) and over 3,000 confirmed infections. **A**. New York City (red curve). **B**. Westchester County (green curve), Nassau County (blue curve), Rockland County (yellow curve) and Suffolk County (purple curve). **C**. Normalized time series showing the number of infected individuals per thousand of county residents, with the same color coding as in panels A and B.

In terms of raw infection counts, the New York City counties taken together transcended all other counties by a factor of at least five, and Rockland County showed the lowest numbers (Fig 5a and 5b). However, in a normalized representation in which the number of infections is reported per 1,000 individuals, New York City comes fourth, following in order Westchester, Rockland and Nassau (Fig 5c). It is also relatively easy to see that confirmed infections at the end of the time window (i.e., on April 1st) do not only show different counts for different counties, but also appear to be increasing at different rates: while Westchester is leading in terms of the proportion of individuals with confirmed diagnoses, Rockland is increasing at a higher rate. In order to better represent the evolution of the rate of change in the context of identifying exponential trends, we again considered the time series in logarithmic form.

In the logarithmic time series, we assessed piecewise linear behavior, starting with the day when the first case was reported until April 1. The slopes *α* (corresponding to the exponential growth rates) and the goodness of fit statistics are shown in Table 3.

**Table 3:** Rate of exponential growth calculated as the slopes of the piecewise linear fit to the logarithmic plot. The sum of squares and Pearson goodness of fit statistic are also provided in each case. (One outlier—March 24—was left out when computing the slopes for Rockland County.).

County	Interval	Slope *α*	Sum of squares	Pearson *χ*^2^
New York City	1-17	0.4345	0.4935	0.0078
	17-22	0.5253	0.1001	0.0128
	22-32	0.1579	0.0911	0.0093
Westchester	7-16	0.1269	0.0116	0.0024
	16-26	0.3239	0.1104	0.0165
	26-32	0.0922	0.0080	0.0009
Nassau	9-18	0.2712	0.1019	0.0256
	18-22	0.5853	0.0462	0.072
	22-32	0.1600	0.0123	0.0015
Suffolk	12-18	0.2744	0.0137	0.0037
	18-22	0.5689	0.0237	0.0041
	22-32	0.1952	0.0543	0.0070
Rockland	9-18	0.2077	0.0747	0.0356
	18-22	0.7036	0.0408	0.0088
	22-32	0.1991	0.0923	0.0123
Saratoga	12-15	0	0	0
	15-21	0.3910	0.1306	0.0629
	21-32	0.1144	0.0829	0.0194
Ulster	12-20	0.1444	0.0618	0.0339
	20-26	0.3275	0.0086	0.0029
	26-32	0.1641	0.0254	0.0050

**Fig 6:**
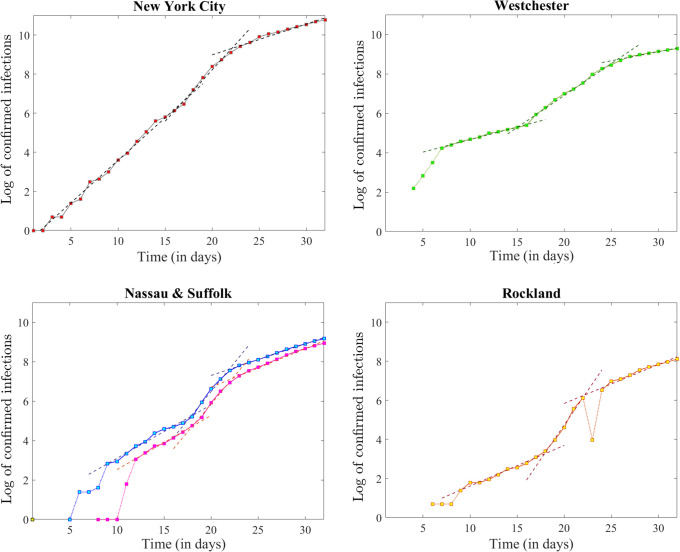
Logarithmic time series corresponding to the data shown in Fig 5, together with the best linear fit (shown as a dashed curve in each case): For the New York City population (top left panel), Westchester (top right), Nassau and Suffolk (blue and respectively purple curves in the bottom left panel), Rockland (bottom right panel).

Notice that all these counties show, via negligible fluctuations, a similar piecewise linear pattern as that of statewide logarithmic time series (see Fig 6). In each case, we were able to identify three pieces: a milder increasing segment ending between March 16-18, followed by a steeper segment ending on March 22 (26 in the case of Westchester), followed again by a linear segment with more relaxed slope, and significant goodness of fit.

**Fig 7:**
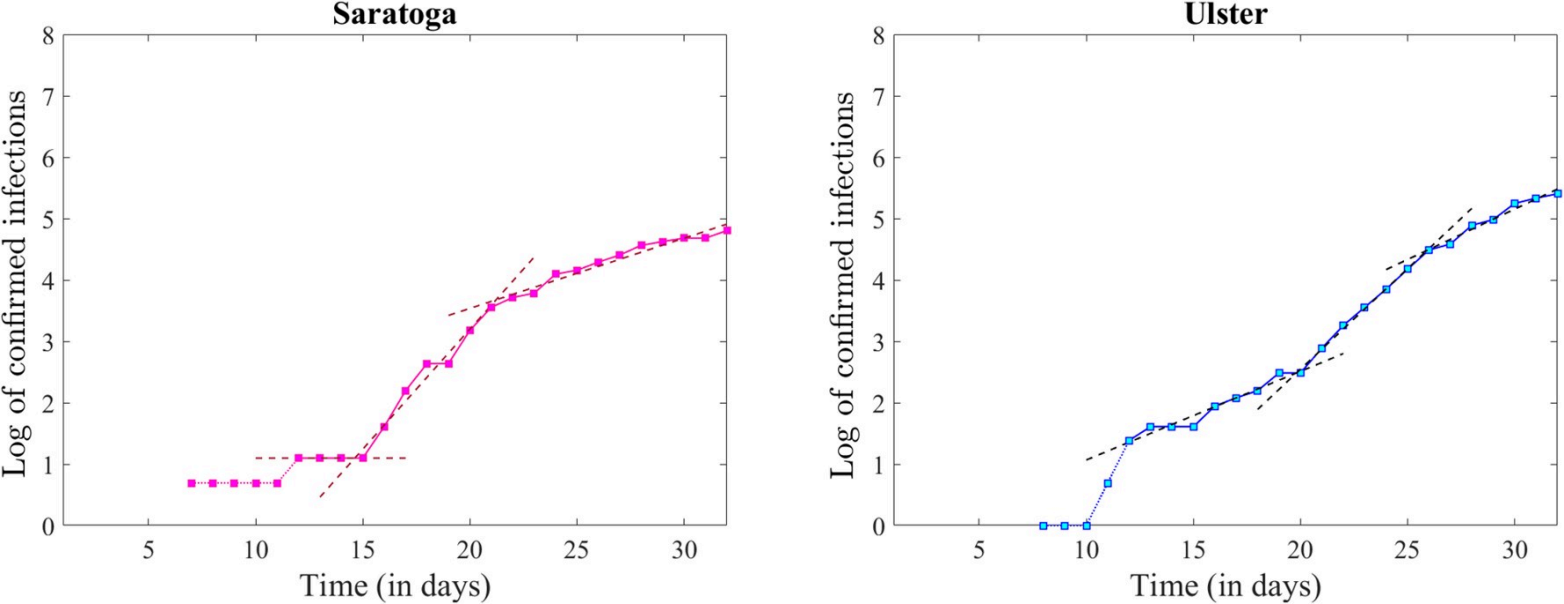
Logarithmic time series together with the best linear fit (shown as a dashed curve in each case): For Saratoga County (left) and Ulster County (right).

While the confirmed infection counts were an order of magnitude lower for Saratoga and Ulster Counties, the same three-piece pattern was apparent in their time series as well (Fig 7), suggesting that the piecewise linear effect with alternating steepness which we observed in the state wide dynamics was not an averaging effect, driven by the counties with high-density urban and suburban areas, but is rather present in every one of the counties with early infections.

We want to further investigate whether the transitions between linear pieces depend on the original confirmed infection in the corresponding county as temporal reference, or if they are synchronized (which would suggest the presence of a statewide unifying factor controlling either the epidemic or the reporting accuracy). While the counties analyzed so far had similar times of original infection, we now proceed to compare the results with those from counties with a delayed timeline (with the first infection confirmed after March 10).

**Fig 8:**
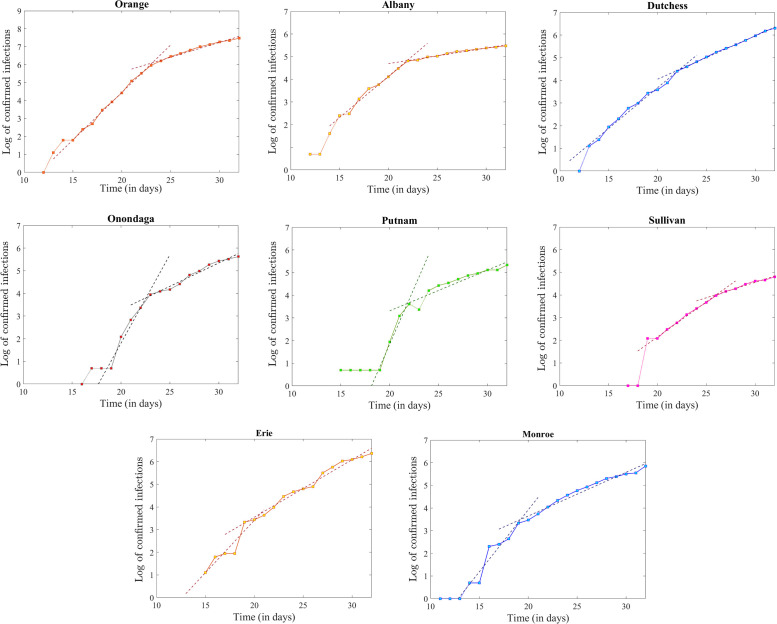
Logarithmic time series together with the piecewise linear fit (plotted as dashed curves in each case) for Orange, Albany, Dutchess, Onondaga, Putnam, Sullivan, Erie and Monroe counties (each shown in a separate panel).

Only eight additional New York State counties had over 100 confirmed infections by April 1, as follows (ordered by total infection count): Orange (1,756), Erie (582), Dutchess (547), Monroe (349), Onondaga (277), Albany (240), Putnam (207) and Sullivan (121). Their logarithmic time series, together with the piecewise linear fits, are shown in Fig 8; the slopes and goodness of fit statistics are described in Table 4. The unifying feature for these counties is the presence of two linear segments (the first with steeper slope, followed by one with flatter slope) with the transition occurring over the same short time window (March 22-26) as the similar transition in the counties with early starts. This confirms that the evolution in this later set of counties is not restarting and replicating the evolution of the earlier counties, but rather is correlating with their current evolution at date (suggesting that the reason behind these trends is a statewide factor rather than an intrinsic epidemic development, as further discussed in the next section).

**Table 4:** Rate of exponential growth calculated as the slope of the piecewise linear fit to the logarithmic plot, for the counties in Fig 8. The sum of squares and Pearson goodness of fit statistic are also provided in each case.

County	Interval	Slope *α*	Sum of squares	Pearson *χ*^2^
Orange	15-23	0.5293	0.0527	0.0155
	23-32	0.1655	0.0499	0.0074
Albany	16-22	0.3640	0.0810	0.0268
	22-32	0.0688	0.0116	0.0023
Dutchess	13-22	0.3583	0.1050	0.0439
	22-32	0.1913	0.0073	0.0014
Onondaga	20-23	0.6149	0.0108	0.0042
	23-32	0.2061	0.0850	0.0174
Putnam	19-22	0.9898	0.1477	0.0707
	22-32	0.1566	0.1164	0.0271
Sullivan	20-26	0.3092	0.0113	0.0042
	26-32	0.1366	0.0105	0.0024
Erie	15-19	0.4621	0.4982	0.2044
	19-32	0.2537	0.2827	0.0553
Monroe	14-19	0.5465	0.6212	0.4150
	19-32	0.1928	0.2246	0.0490

## 4 Discussion

In this study, we focused on the dynamics of the COVID-19 epidemic in the state of New York for the first month of the outbreak (March 1st to April 1st), and we analyzed statewide data on confirmed infections, testing, hospitalizations and deaths, as well as county-wise data on infection rates, for the counties in the state that were reporting over 100 infections by April 1. Our primary goal was to determine whether it is possible to dissociate between intrinsic trends in the epidemic dynamics, the effects of social distancing measures, and the effects of under-reporting produced by the schedule and limitations in testing.

We first identified a signature in the evolution of the number of confirmed infections specific to the state of New York, characterized by piecewise behavior with four distinct pieces, switching between exponential curves with higher versus lower alternating rates, and eventually settling to a relatively low exponential rate. We aimed to understand the factors behind the transitions, with a primary focus on answering whether the trend shown in the last piece of the data (preceding the start of April) corresponds to a damping in infections, or is a reporting artifact.

Analyzing data on statewide testing, we found that, while the number of tests steadily increased, the ratio of positive to total tests also increased before March 12, to the point where over 70% of tests were positive. This gradual saturation could be indicative of two potential factors: the infection rate overtook the rate with which new tests were becoming available; or the limited testing was particularly targeted to individuals with highest probability of being infected (well-defined COVID-19 symptoms), dangerously leaving out other infected, but less symptomatic individuals.

We detected a noticeable jump in the number of tests on March 13th, (which could be attributed to the state government directive on March 12 to ramp up COVID-19 testing), and a subsequent drop in the positive test percentage from 70% to under 15%. The time of the first rate increase found in the confirmed-infection time series occurred shortly after this (March 16). A likely interpretation is that the increasingly wider availability of tests led to an increased detection rate, and subsequently to more accurate reporting. For specific counties, the duration of this transition in the slope ranged from March 12 to March 18, but can still be viewed as a potential effect of the increase in testing, as the factor synchronizing the piecewise behavior across county borders. This is supported by the fact that the counties with later infection onset do not show this transition, and start off with a segment of higher exponential rate directly. We therefore suggest that the higher confirmed infection rate during the period March 16—March 23 reflects the actual epidemic spread more faithfully than the rates along the periods preceding and following it (when tests were administered more conservatively). After March 13, the positive test percentage promptly started increasing again, having climbed back to almost 40% by April 1. The subsequent climb of this ratio could be explained by the rate of increase in actual infections transcending the rate of increase in testing, thus slowly saturating again the testing capacity.

We also detected a statewide transition in the confirmed infections on March 23, from a higher exponential rate *α* ∼ 0.46 to a lower rate *α* ∼ 0.15. The subsequent analysis revealed that all counties showed a similar transition at dates spanning from March 19 to March 23, from a higher exponential rate (with mean *μ*_*α*_ = 0.51 and standard deviation *σ*_*α*_ = 0.18 between the 15 counties examined) to a milder rate (with mean *μ*_*α*_ = 0.16 and standard deviation *σ*_*α*_ = 0.04). As mentioned previously, there are two potential (and not mutually exclusive) factors that could be held responsible for this behavior: testing restrictions and social distancing. Below, we discuss both possibilities.

One possibility if that the lower infection rate may be a reflection of the stricter testing cap, re-introduced throughout the state of New York around March 20, to address PPE and other resource shortage. The number of actual infections was increasing statewide at an exponential rate close to *α* ∼ 0.5 prior to March 23, to the point of overtaking the testing capacity (which was following a more relaxed exponential curve). In order to maintain realistic reporting, testing would have had to also increase at a comparable pace. The March 20 recalibration of testing priorities likely resulted in restricting testing of both positive (but potentially asymptomatic) and negative COVID-19 individuals to the same extent (people being instructed to refrain from testing unless in very serious condition). While there was no visible drop in test numbers, or additional imbalance in positive test percentage, the testing restriction may have effectively capped the reported infections. This would explain why the rate of the new infections (*α* = 0.15) settled to a rate comparable to the overall rate of testing (*α* = 0.115), likely reflecting more the slower rate of the testing cap rather than the potentially much faster rate of infections.

If the resulting lower exponential rate over the period from March 23 to April 1 is indeed only an artifact of the testing cap, we can estimate a more realistic number of infections on April 1, by considering the higher infection rate *α* = 0.46 shown prior to March 23 to remain in effect until April 1. The estimate of the total number of infections in New York State by April 1 would then be 1,302,800. This should be compared to the reported 83,712, and the projected 80,524 (when using the *α* = 0.15 lower rate to extend). This is in line with reports from a preliminary serological study that estimates actual infections many times higher than the confirmed infection counts, indicating a 11-18% prevalence of COVID-19 infections in New York State residents (with wide geographic variation) [24, 25]. However, the accuracy of serological findings is also problematic, with sampling biases such as false positives and test performance issues potentially affecting the results.

A second possibility worth investigating is that the rate flattening in all counties between March 19-23 represents an effect of social distancing. The timing of this change is too premature to reflect any effects of the PAUSE directive, or even of the state-mandated initial closures initiated between March 12 and 16 (which would need around two weeks to manifest significantly). But this does not exclude the possibility of people having initiated social distancing before it was required at state level. One way to assess whether this aspect had any contribution to the infection dynamics is to search for decreasing patterns in social mobility, and verify if the drop in infection rate correlates, with an appropriate time lag, with a drop in mobility.

Social mobility trends can now be estimated based on data made available by both Google and Apple, describing traffic patterns (driving, walking and public transport), as well as number of direction requests to different types of destinations (Retail/Recreation, Grocery/Pharmacy, Parks, Public Transit, Workspace), and time spent at one’s Residence. A broad assessment of this data suggests that, across New York State counties, mobility patterns did not start showing consistent decreasing patterns with respect to their respective baselines until the second week in March, with an inflection point around March 15, and a minimum consistently reached around the date of the PAUSE directive. Hence early social distancing does not seem to offer a compelling basis for the infection rate decline observed in the clinical data. In a subsequent study, we focus on addressing further potential correlations between mobility measures and epidemic measures, in longitudinal data spanning a timeline from March 1 until June 21 [26]. The study significantly correlates peak infections and epidemic outcome with the timing and degree of reduction in social mobility, and suggests that longer lags of up to 40 days may be more meaningful when assessing the full impact of social distancing on epidemic size and dynamics.

### 4.1 Limitations and future work

It is important to discuss some constraints imposed on our study by the length, quality and accessibility of data in the public domain. An inherent problem comes from the length of the time series for confirmed infections (only spanning one month, with infection in many of the counties having been ongoing in fact for much shorter than that). Aside from the analytical concern associated with detecting trends in short time series, the time period was insufficient to permit assessment of the effects of the PAUSE directive, or indeed of any social distancing. A second limiting aspect is the fact that some of the measures of interest (such as hospitalizations and deaths) were not included promptly or accurately in the preliminary reports. This is partly due to the priority assignment during the early stage of the outbreak, and partly due to the fact that assessment of certain measures may require time and hindsight. For example, data on hospitalizations only started being included in the public domain repository on March 20, and was not separated by county in the original reports, nor was the statewide number of available tests. The original identification criteria of COVID-related deaths had to be later redefined to better incorporate progressing knowledge on the clinical aspects. The death count, expected by many to be a more accurate epidemic measure than confirmed infections, had to be subsequently updated by an additional 3,000 individuals.

On one hand, these limitations are significant; on the other hand, however, they go together with the very necessity of data analyses in the *early* stages of the epidemic, so that it can be used to understand its dynamic signature, generate testable predictions, and apply the appropriate measures *before* the outbreak gets out of control and transcends the health care capacity. While longer, more complete and informed data streams would likely improve predictions, waiting for more complete data may concomitantly have a detrimental impact on the timeliness of the response [27].

Along these lines, social mobility time series that were not available during the first weeks of COVID-19 development in the New York State have been made easily accessible in the public domain by Google and Apple. This prompted a follow-up study, in which we revisited the New York State data, with an improved perspective based on (1) a longer timeline (covering the period starting with the March 1 initial confirmed infection and extending until the last week of June); (2) access to social mobility data, as a quantifiable measure of the efficiency of lockdown and social distancing measures in each New York county [26]. Our study is detecting county-wise correlations between the number of confirmed infections, COVID-related hospitalizations and deaths, and effectiveness of social measures along the closing down, PAUSE, and reopening of the state. While the initial slope flattening in the current study appears to be primarily an effect of testing limitations, our follow-up study suggests that social distancing measures can be identified further down the line as a primary player in controlling the outbreak in New York State.
